# The potential of diallyl trisulfide for cancer prevention and treatment, with mechanism insights

**DOI:** 10.3389/fcell.2024.1450836

**Published:** 2024-09-30

**Authors:** Ling Lu, Zihan Gao, Jiajia Song, Longtao Jin, Zhaofeng Liang

**Affiliations:** ^1^ Child Healthcare Department, The Fourth Affiliated Hospital of Jiangsu University, Zhenjiang, China; ^2^ Jiangsu Key Laboratory of Medical Science and Laboratory Medicine, School of Medicine, Jiangsu University, Zhenjiang, China

**Keywords:** diallyl trisulfide, cancer, prevention, treatment, mechanism

## Abstract

Cancer has become an important public health problem worldwide, and there is currently a lack of effective treatment and prevention strategies. Natural plant active ingredients have been proven to be a safe and highly promising method for preventing and treating cancer. It has been found that diallyl trisulfide have anticancer effects in multiple types of cancer via inhibiting cancer proliferation, enhancing chemotherapy sensitivity, inducing apoptosis/autophagy, suppressing invasion/migration, regulating microenvironment. With the deepening of research on new strategies for cancer prevention and treatment, the role of diallyl trisulfides in cancers occurrence, prognosis, and drug resistance is also receiving increasing attention. In order to better understand the relationship between diallyl trisulfides and various cancer, as well as the role and mechanism of diallyl trisulfides in cancer prevention and treatment, we briefly summarized the role and function of diallyl trisulfide in cancers.

## 1 Introduction

Because of its high incidence rate and mortality, cancer has gradually become a serious public health problem endangering life and health worldwide (Siegel et al., 2024). Despite the continuous advancement of cancer diagnosis and treatment technology, the effective rate of cancer treatment is still very low, resulting in a high recurrence rate and poor prognosis of cancer. Therefore, it is necessary to search for new interventions or treatment strategies for cancer. The development of new anti-cancer drugs is extremely challenging and time-consuming. On the other hand, due to the serious side effects of treatment methods such as chemotherapy and radiation therapy, it increases the burden and makes the treatment of cancer patients more challenging (Sohel et al., 2022). More and more evidence suggest that exercise and changing dietary habits may effectively prevent and play an important role in tumor treatment. Over the years, natural products have become increasingly popular in clinical settings. Natural products, such as plants, animals, and microorganisms, account for approximately 75% of clinical use of anticancer drugs, and have been used in medicine for decades, typically as supplements and substitutes (Sohel et al., 2022; Newman and Cragg, 2016). Among numerous natural products and dietary factors, phytochemistry is gradually showing their importance in anti-tumor research because they have many advantages such as wide sources, excellent anti-cancer effects, high safety, and ease of application.

**FIGURE 1:**
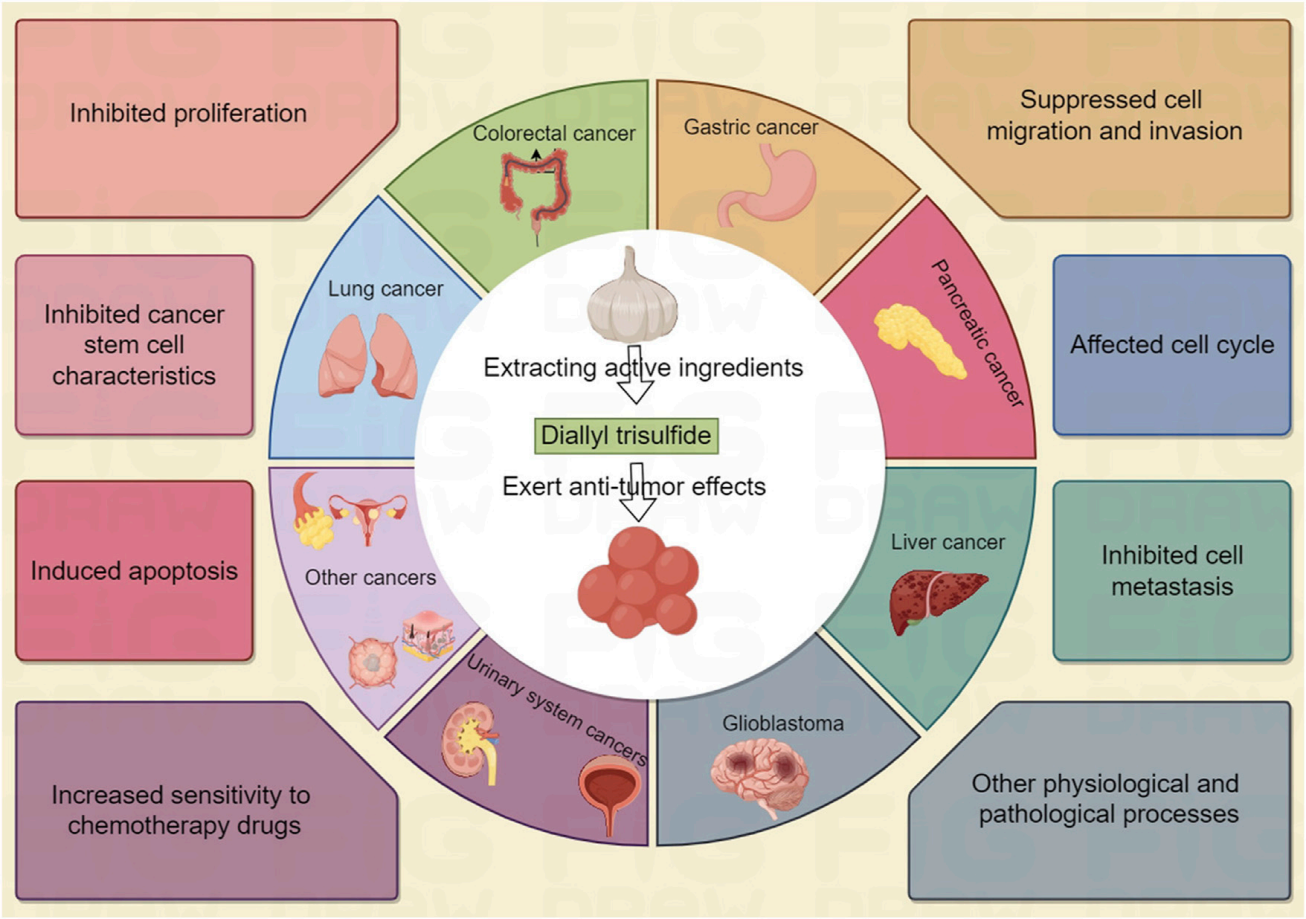
By Figdraw. The role and molecular mechanisms of DATS in cancer treatment and prevention.

Currently, phytochemicals are the main candidate drugs most suitable for developing anti-cancer drugs (Malla et al., 2022). Studies have shown that natural products such as Diallyl trisulfide (DATS) selectively act on cancer cells by targeting multiple pathways related to cancers, with minimal impact on normal cells (Malla et al., 2022; Khan and Uddin, 2021). Allium plants, such as garlic and onions, have long been considered to have medicinal value (Puccinelli and Stan, 2017; Zhang et al., 2024). Many studies have shown that DATS is a biologically active organic sulfur compound found in garlic, which can regulate disease states such as cancer, infection, metabolic syndrome and various other diseases (Zhang et al., 2024; Rauf et al., 2022; Mondal et al., 2022). In this review, we summarize the role and molecular mechanisms of DATS in cancer treatment and prevention (Figure 1), and point out the shortcomings of existing research and challenges for future clinical applications.

## 2 The interventional effect and mechanism of DATS in cancer

### 2.1 DATS intervention in breast cancer

**FIGURE 2:**
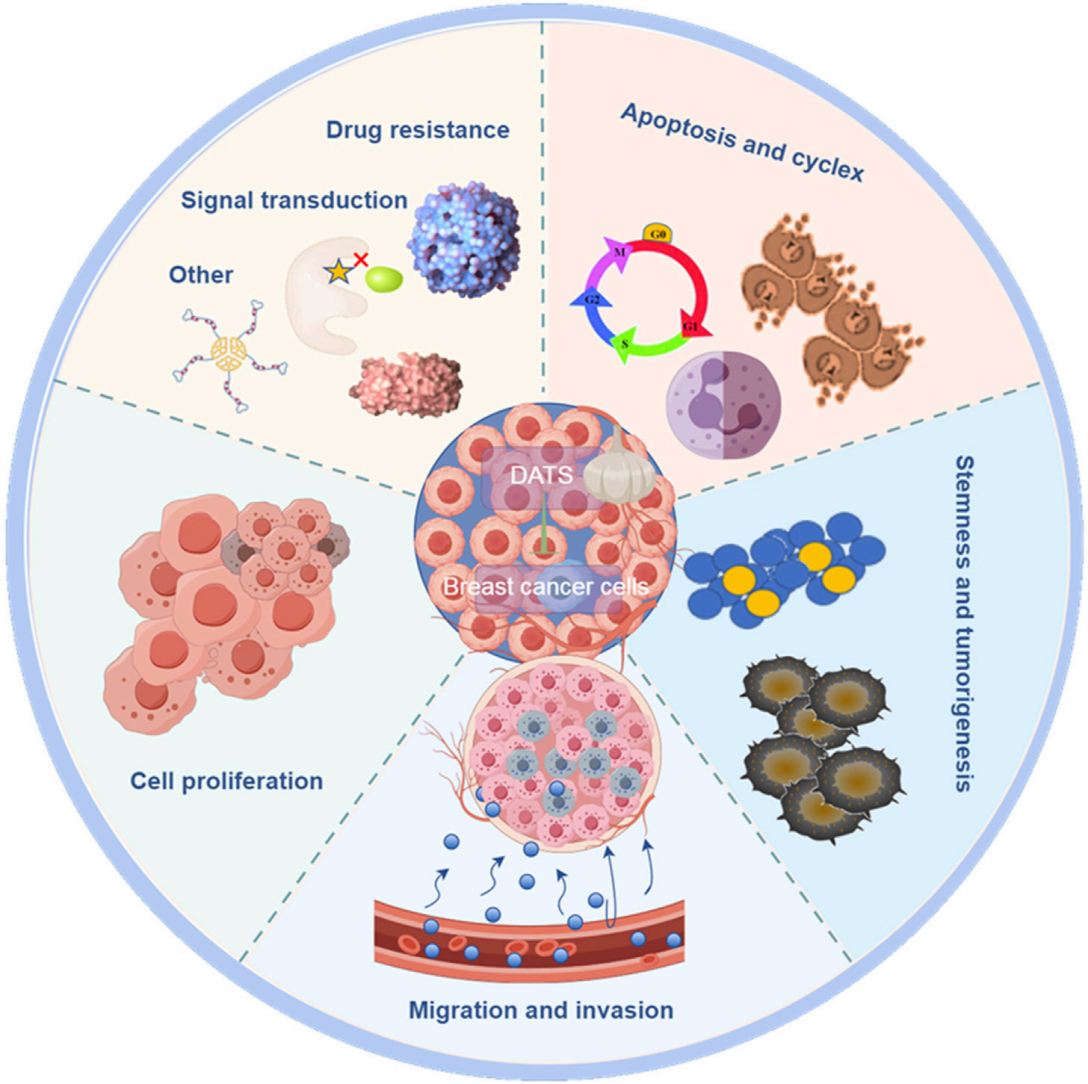
By Figdraw. The anticancer effect of DATS in breast cancer.

Breast cancer is one of the major causes of cancer related deaths among women worldwide (Malla et al., 2022). In this section, we discussed the potential anticancer activity of DATS in breast cancer, focusing on the intervention and treatment on proliferation, invasion, drug resistance cell cycle arrest, apoptosis, stem cell specificity and other aspects of breast cancer (Table 1; Figure 2). Understanding the anticancer activities of DATS provides insights into their potential in targeting the mechanism of occurrence and development of breast cancer.

**TABLE 1:** Overview of the effect of DATS on breast cancer.

Effects	Target	Subjects	References
Inhibited proliferation, ROS formation and AhR expression	ARNT/HIF-1β, CYP1A1, DNA POLβ	Breast cancer cells	Ferguson et al. (2024b)
Suppressed cell migration	Dynamin-1-like protein	Breast cancer cells	Hahm et al. (2022)
Affected cell cycle	LIT/ROBO	Breast cancer cells	Hahm et al. (2021)
Affected cell cycle and induced apoptosis	NF-κB and MAPK	Breast cancer cells	Kanga et al. (2023)
Induced apoptosis and inhibited cell proliferation	Cytochrome c and caspases	Breast cancer cells	Stan and Abtahi (2022)
Inhibited cancer stem cell characteristics	Wnt/β-catenin	Breast cancer stem cells	Li et al. (2018)
Inhibited cancer stem cell characteristics	Monocarboxylate transporter 1	Breast cancer stem cells	Kim and Singh (2022)
Inhibited cancer stem cell characteristics	Forkhead box Q1	Breast cancer stem cells	Kim et al. (2016)
Inhibited cell proliferation	Notch pathway	Breast cancer cells	Kiesel and Stan (2017)
Reduced inflammation, proliferation, enhanced apoptosis	Notch pathway	Breast cancer cells	Elsherbiny et al. (2020)
Inhibited cell proliferation, migration and invasion	Leptin/STAT3	Breast cancer cells and rats	Kim et al. (2020)
Induced apoptosis and inhibited cells viabilities	ER-α/Pin1	Breast cancer cells	Hahm and Singh (2014)
Increased sensitivity to chemotherapy drugs	\	Breast cancer cells	Marni et al. (2022)
Inhibited the proliferation and migration	Lactate dehydrogenase A	Breast cancer cells	Cheng et al. (2017)
Induced cell death and inhibited cell migration	CCL2	Breast cancer cells	Kanga et al. (2021)
Attenuated progression and metastasis	NF-κB, MMP2/9, Trx-1	Breast cancer cells	Liu et al. (2018)
Inhibited cell metastasis	NF-κB and ERK/MAPK	Breast cancer cells	Liu et al. (2015)
Suppressed the hematogenous metastasis	PAF	Breast cancer cells	Liu et al. (2017)
Reduce the hematogenous metastasis	HIF-1 α	Breast cancer cells	Wei et al. (2017)

DATS inhibited proliferation, ROS formation, 8-OHdG levels, AhR expression and clonogenic formation in breast cancer cells compared to B [a] P group (Ferguson et al., 2024a). Diallyl trisulfide plays an anti-cancer role by reducing B [a] P induced oxidative stress, AhR expression and DNA damage in human breast cancer epithelial cells (Ferguson et al., 2024b). These results provided evidence for the intervention of DATS in breast cancer. Hahm et al. (2022) research found that actin cytoskeleton is a new target of DATS in SK-BR-3 cells, which may explain its inhibitory effect on migration of breast cancer cells. As is well known, cell cycle arrest plays a crucial role in the research of anti-tumor drugs. DATS regulated LIT/ROBO tumor suppressor signal related genes, thereby affecting cell cycle to play the role of anti-breast cancer (Hahm et al., 2021). DATS regulated cell cycle arrest, influenced caspase activity, induced apoptosis of breast cancer cells to play an intervention role (Kanga et al., 2023). Stan et al. showed that DATS inhibited proliferation and induced apoptosis of breast cancer cells, suggesting that DATS may be a potential cancer preventive agent (Stan and Abtahi, 2022). Breast cancer stem cells play an important role in the regulation of breast cancer. The research results of Li et al. (2018) indicate that DATS inhibits the Wnt/β-catenin pathway to inhibit the activities of breast cancer stem cells may provide a new strategy for the prevention of breast cancer. Kim and Singh (2022) also found that DATS suppress stem cell stemness of breast cancer and monocarboxylate transporter 1 can be a new target. These results of another study indicated that forkhead box Q1 is a new target for DATS to inhibit the stemness of breast cancer stem cells (Kim et al., 2016). DATS inhibits the expression of ADAM10 and ADAM17 by regulating Notch signaling pathway, and plays an intervention and therapeutic role in breast cancer (Kiesel and Stan, 2017). Elsherbiny et al. (2020) pointed out that the anti-cancer effect of DATS is mediated by inhibiting Notch signaling protein, reducing tumor inflammation, proliferation, and enhancing cell apoptosis. DATS suppressed leptin induced tumor signaling in breast cancer cells, thereby inhibiting proliferation, survival, migration and invasion of cells (Kim et al., 2020). DATS could induce apoptosis and inhibit cells viabilities by inhibiting estrogen receptor-α activity of breast cancer cells (Hahm and Singh, 2014). These data of this research indicated that DATS may be a safer alternative treatment scheme targeting estrogen receptor-α, and it has great application prospects in the prevention of breast cancer. DATS inhibited the cell proliferation of PTX resistant breast cancer cells by blocking cell cycle and inducing apoptosis, thereby affecting the drug sensing effect of breast cancer cells (Marni et al., 2022). DATS has an inhibitory effect on the Warburg effect in breast cancer cells, but has no significant effect on normal cells. DATS inhibits the cell proliferation and migration by downregulating the activity of lactate dehydrogenase A in breast cancer cells (Cheng et al., 2017). Warburg effect plays a crucial role in the occurrence of breast cancer and other cancers. Exploring specific drugs such as DATS targeting key proteins in the Warburg effect, may be a very promising strategy for treating cancer. Migration and invasion are two key steps of cancer metastasis, including breast cancer. Blocking the migration and invasion of cancer cells may be an effective strategy to reduce the risk of breast cancer, especially triple negative breast cancer. DATS reduced the expression of CCL2 in TNF-α-treated MDA-MB-231 cells, inducing cell death and inhibiting cell migration (Kanga et al., 2021). It is reported that DATS targeting Trx-1 system inhibits metastasis in nude mice inoculated with breast cancer cells, which may provide a promising strategy for the treatment of triple negative metastasis (Liu et al., 2018). Liu et al. showed that DATS inhibits the metastasis of triple negative breast cancer by suppressing NF-κB and ERK/MAPK pathways (Liu et al., 2015). It is found that DATS suppressed the hematogenous metastasis of MDA-MB-231 cells in the presence of platelets activated by PAF (Liu et al., 2017). DATS could also inhibit HIF-1 α synthesis to reduce the hematogenous metastasis of MDA-MB-231 cells (Wei et al., 2017). The hydrophobicity, short half-life, lack of target selectivity and limited bioavailability of DATS at tumor sites limit its efficacy in the treatment of breast cancer, especially triple negative breast cancer. In order to overcome these limitations, researchers have developed folate coupled DATS SLN, which improves the intervention effect of DATS on breast cancer (De et al., 2023; Hahm and Singh, 2022).

As a natural phytochemical, DATS is expected to become a new prevention and treatment strategy for breast cancer, especially triple negative breast cancer, which has great clinical application prospects. DATS play an intervention and therapeutic role in the proliferation, migration, invasion, drug resistance, cell cycle arrest, apoptosis, stem cell specificity and other aspects of breast cancer.

### 2.2 The intervention effect of DATS in gastric and colorectal cancer

Gastric cancer and colorectal cancer are the leading malignant tumors in terms of incidence rate and mortality worldwide. The stomach and colon are the main sites for the digestion and metabolism of phytochemicals such as DATS, so DATS may have good preventive and therapeutic effects on gastric and colorectal cancer (Wang et al., 2022). In this section, we discussed the potential anticancer activity of DATS in gastric cancer and colorectal cancer, focusing on the intervention and treatment on proliferation, recurrence, invasion cancer stem properties and drug resistance of breast cancer (Table 2). These are of great reference value for the search for new prevention and treatment strategies for digestive system cancers such as gastric cancer and colorectal cancer.

**TABLE 2:** Overview of the effect of DATS on gastric and colorectal cancer.

Effects	Target	Subjects	References
Inhibited cancer stem cell characteristics	ΔNp63/SHH	Gastric cancer stem cells	Ge et al. (2023)
Increased sensitivity to chemotherapy drugs	STAT3/PKC-δ/MAPK	Gastric cancer cells and rats	Lv et al. (2023)
Increased sensitivity to chemotherapy drugs	Nrf2/Akt and p38/JNK	Gastric cancer cells and rats	Jiang et al. (2017a)
Increased sensitivity to chemotherapy drugs	NF-κB	Gastric cancer cells and rats	Pan et al. (2016)
Induced apoptosis and inhibited cell proliferation	Cytochrome c and caspases	Gastric cancer tissues and cells	Wang et al. (2019)
Induced apoptosis and mitotic arrest	AMPK pathway	Gastric cancer cells	Choi (2017)
Interacted with the Cys288 residue of Keap1	Nrf2	Gastric cancer cells	Kim et al. (2014)
inhibited tumor growth, migration, invasion and promoted apoptosis	MAPK/cytokines	Gastric cancer cells and rats	Jiang et al. (2017b)
Inhibited cancer stem cell characteristics	Wnt/β-catenin	Colorectal cancer stem cells	Zhang et al. (2018a)
Inhibit the migration, invasion and angiogenesis	Notch pathway	Colorectal cancer cells and rats	Alrumaihi et al. (2022)

Gastric cancer is the common malignant cancer of the digestive system, and most patients are already in the advanced stage when diagnosed. Prevention and effective treatment strategies are particularly important. Gastric cancer stem cells play a crucial role in the progression, recurrence, and drug resistance of gastric cancer. Reported by Ge et al. (2023) that DATS inhibited the properties of gastric cancer stem cells via ΔNp63/SHH axis, exerting an intervention effect in gastric cancer and providing strategies for the prevention and treatment of gastric cancer. The research results indicate that the combination of DATS and cisplatin regulates endoplasmic reticulum stress and inhibits STAT3/PKC-δ collaborate with the MAPK pathway to enhance anti-tumor activity (Lv et al., 2023). DATS suppressed gastric cancer growth and enhanced the efficacy of cisplatin by inhibiting Nrf2/Akt and activating p38/JNK pathway (Jiang X. Y. et al., 2017). Epigenetic upregulation of metallothionein 2A by DATS through inhibition of NF-κB activation and enhancement the chemical sensitivity of gastric cancer cells to paclitaxel (Pan et al., 2016). Resistance to chemotherapy drugs is a very challenging task in cancer treatment. DATS can enhance the therapeutic effect of cisplatin, cisplatin, docetaxel and reduce cell toxicity, making it an ideal potential treatment option for gastric cancer patients. Sulfidoxine is a newly discovered antioxidant enzyme that is overexpressed in various cancers and may promote the occurrence and progress of gastric cancer. After treatment with DATS, the expression of sulfidoxine, MDA level, and ROS level in BGC823 cells were significantly inhibited, and DATS may exert anti-cancer effects by regulating sulfidoxine (Wang et al., 2019). It is reported that DATS induces gastric cancer cells apoptosis and mitotic arrest through activation of AMP activated protein kinase mediated by reactive oxygen species (Choi, 2017). These findings by Kim et al. (2014) suggested that DATS can interact with the Cys288 residue of Keap1, which to some extent explains its ability to induce Nrf2 activation and upregulate defense gene expression, playing an intervention role in gastric cancer. Jiang et al. (2017b) demonstrated that DATS exerts anti-gastric cancer effects in SGC-7901 tumor bearing mice by activating the MAPK pathway and regulating cytokines.

More and more evidence show that DATS has excellent intervention and therapeutic effects in multiple processes and pathological processes of gastric cancer occurrence and development, providing new strategies for early prevention and treatment of gastric cancer and enhancing chemotherapy efficacy.

Colorectal cancer is the third most common malignant cancer worldwide, with a high mortality rate due to treatment failure and cancer recurrence, with a 5 years relative survival rate of only 8%. Exploring the role of phytochemicals such as DATS in the intervention and treatment of colorectal cancer can provide new evidence for clinical prevention and treatment (Oravetz et al., 2023). Colorectal cancer stem cells play a crucial role in the initiation, development and metastasis of colorectal cancer (Liao et al., 2023). The results of Zhang et al. demonstrated that Wnt/β-catenin pathway mediates the inhibitory effect of DATS on colorectal cancer stem cells, playing an intervention and therapeutic role in colorectal cancer (Zhang Q. et al., 2018). Report has shown that DATS can inhibit the angiogenesis, migration, invasion of colon cancer cells, and can also inhibit the growth of transplanted tumors in nude mice. In order to enhance the anti-colorectal cancer effect of DATS, researchers constructed a lipid-based nanoparticle normula of DATS, which improved the anti-colorectal cancer effect of DATS (Alrumaihi et al., 2022). In summary, the above research results indicate that DATS is an effective anti-tumor phytochemistry and may become a clinically useful drug for the treatment of human colorectal cancer.

### 2.3 The preventive and therapeutic effects of DATS in gliomas

Gliomas is one of the most malignant brain tumor. Although multimodal treatment for glioblastoma includes surgery, radiotherapy, and chemotherapy, the extension of survival for gliomas patients is still limited. Therefore, there is an urgent need to find new strategies to improve the therapeutic efficacy of gliomas. DATS has shown excellent intervention effects in various cancers including gliomas (Choromanska et al., 2020). In this chapter, we summarize the preventive and therapeutic effects of DATS in gliomas (Table 3).

**TABLE 3:** Overview of the effect of DATS on gliomas and lung cancer.

Effects	Target	Subjects	References
Enhanced ROS accumulation, apoptosis, DNA damage, as well as inhibition of tumor growth of GBM cells	Cysteine 32/35 and thioredoxin 1	Glioma cells and animal models	Tian et al. (2022)
Regulated cell proliferation and antioxidative effects	Bcl-2/MPST and rhodanese	Glioma cells	Jurkowska et al. (2017)
Inhibited proliferation, invasion and angiogenesis	Wnt/β-catenin	Glioma cells and nude mouse	Tao et al. (2017)
Prevented tumor progression and induced apoptosis	Histone acetylation and caspase-3	Glioma cells and PDX animal models	Das et al. (2018)
Enhanced apoptosis	TRAIL/death receptor 5/Mcl-1	Glioma cells	Hwang et al. (2017)
Inhibited LPS induced inflammatory response	NF-κB/Toll-like Receptor 4 and CXCL12/CXCR4	Glioma cells	Lee et al. (2018)
Inhibited the CSCs activity	Wnt/β-catenin	Human bronchial epithelial cells	Wang et al. (2018)
Modulate gut microbiota	PPARγ/NF-κB pathway	A/J mice	Qu et al. (2024)
Inhibited cell growth and improved cisplatin induced oxidative damage	Caspase-3/8/9 and JNK/p38 MAPK pathways	Lung cancer cells and Xenograft Mice	Jiang et al. (2017c)
Affected iCpGI demethylation	UHRF1/DNMT1-MZF1 axis	Lung cancer cells and patients	Lin et al. (2023)
Induced pyroptosis and impaired lung CSC-like properties	ROS/Caspase 1	Lung cancer cells and Xenograft Mice	Xie et al. (2024)

It is demonstrated that DATS sensitizes radiation therapy on glioblastoma through directly conjugates with cysteine 32/35 and targeting thioredoxin 1 (Tian et al., 2022). The unlimited proliferation of cancer cells is one of the main causes of cancer progression. The inhibition of glioma cells proliferation by DATS is associated with an increase in the inactivation form of Bcl-2 (Jurkowska et al., 2017). The research results of Tao demonstrated that DATS inactivate Wnt/β-catenin pathway inhibits the cell proliferation, angiogenesis and invasion of glioma cells (Tao et al., 2017). DATS regulates an increase in histone acetylation and activation of caspase-3, leading to a dose-dependent reduction in total tumor volume, proliferation, and angiogenesis in glioma (Das et al., 2018). It is reported that DATS upregulates death receptor 5 through ROS, making glioma cells sensitive to TRAIL mediated apoptosis (Hwang et al., 2017). These results of Lee indicated that DATS inhibits the activation of the CXCL12/CXCR4 axis associated with TLR4 antagonism and blocks NF-κB signal transduction, thereby demonstrating anti-inflammatory effects to intervene in glioma (Lee et al., 2018). DATS is an organic sulfur compound derived from garlic, which has been proven to have potential anti-inflammatory, anti-proliferative, angiogenic, enhanced radiation and other therapeutic effects.

### 2.4 The role of DATS in lung cancer

Lung cancer is the leading cause of cancer-related deaths worldwide, and there is currently a lack of effective and minimally toxic therapies in clinical practice. Researchers are increasingly focusing on the preventive and therapeutic effects of phytochemicals such as DATS on lung cancer (Table 3).

Wang et al. reported that DATS regulate the activity of the Wnt/β-catenin pathway modulates the acquisition of cancer stem cell characteristics induced by chronic tobacco smoke exposure (Wang et al., 2018). The results of Qu et al. indicated that DATS significantly decreased the number of lung tumors in A/J mice induced by 4-(methylnitrosamino)-1-(3-pyridyl)-1-butanone, which may be related to DATS regulating the gut microbiota, especially increasing the abundance of *F. rodentium* (Qu et al., 2024). It is reported that DATS regulates JNK and p38 pathway to inhibit the growth of NCI-H460 and improve the therapeutic effect of cisplatin induced oxidative damage on xenograft tumor mice and cells (Jiang et al., 2017c). The synergistic effect of DATS and 5-aza-2 ′- deoxycytidine allows for site-specific iCpGI demethylation of PRSS3-V3 upregulated by MZF1, exerting anti-cancer effects in lung adenocarcinoma cells (Lin et al., 2023). The research results of Xie et al. (2024) suggested that DATS promotes cell pyroptosis and attenuates lung cancer stem cells properties by activating the ROS/Aspase 1 signaling pathway, thereby delaying the progression of lung cancer.

At present, there is relatively little research on the prevention and treatment of DATS in lung cancer, and there are many types of lung cancer with different pathogenesis. In future research, it is necessary to observe the preventive and therapeutic effects of DATS on different types of lung cancer or the main risk factors for lung cancer, such as tobacco exposure.

### 2.5 The preventive and therapeutic effects of DATS in the urinary system

DATS played an intervention role in smoke induced bladder cancer by regulating NF-κB pathway to inhibit the cancer stem cell characteristics and EMT (Table 4) (Geng et al., 2021). DATS induced apoptosis of bladder cancer cells to play an intervention effect, and PI3K/Akt and JNK pathways play a regulatory role in this process (Shin et al., 2014). DATS is a fully characterized H2S donor that inhibits the proliferation of urothelial carcinoma cells and promotes cell apoptosis in a time and concentration dependent manner (Panza et al., 2022). DATS mediated the inhibition of cancer cell proliferation, invasion and migration of bladder cancer. ANGPTL4 could regulate the intervention effect of DATS, which may become a prognostic marker of bladder cancer patients (Shin et al., 2017).

**TABLE 4:** Overview of the effect of DATS on urinary system and osteosarcoma.

Effects	Target	Subjects	References
Inhibited the cancer stem cell characteristics and EMT	NF-κB pathway	Bladder animal models	Geng et al. (2021)
Induced cell apoptosis	PI3K/Akt and JNK pathways	Bladder cancer cells	Shin et al. (2014)
Inhibited the proliferation and promoted apoptosis	H2S pathway	Urothelial carcinoma cells	Panza et al. (2022)
Suppressed proliferation, migration, and invasion	ANGPTL4	Bladder cancer cells	Shin et al. (2017)
Overcomed apoptosis resistance and induced ferroptosis	—	Prostate cancer cells	Samy et al. (2022)
Inhibited the renal CSC-like properties	Nanog	Renal cancer cells	Zhang et al. (2023)
Induced cell apoptosis	PI3K/Akt pathway	Osteosarcoma cells	Wang et al. (2016)
Suppressed cell invasion, migration and proliferation	PPARγ/NF-κB pathway	Osteosarcoma cells and Xenograft Mice	He et al. (2020)
Inhibited cell growth, migration, EMT and induced apoptosis, autophagy	EGFR/PI3K/AKT/mTOR pathway	Osteosarcoma cells	Liu et al. (2024)
Inhibited cell growth	CRT	Osteosarcoma cells	Xie et al. (2018)
Inhibited cell growth	GRP78	Osteosarcoma cells	Zhang et al. (2018b)
Induced cell apoptosis and reduced multidrug resistance	NF- κ B	Osteosarcoma cells	Wang et al. (2014)

Apoptosis resistance is a potential mechanism for drug resistance in prostate cancer treatment. DATS effectively targeted prostate cancer cells by overcoming cell apoptosis resistance and inducing prostate cancer cell death mediated by ferroptosis (Samy et al., 2022). Cancer stem-like cells play an important role in cancer such as renal cancer initiation and progression. DATS inhibited the renal cancer stem-like cell properties by suppressing Nanog (Zhang et al., 2023).

### 2.6 Prevention and treatment of osteosarcoma

Osteosarcoma is the most common non hematopoietic primary bone cancer, mainly occurring in young people and adolescents (Table 4). At present, the preferred treatment for osteosarcoma includes neoadjuvant chemotherapy followed by surgery and chemotherapy, but most patients are prone to recurrence. We urgently need to discover new natural compounds such as DATS that have the potential to prevent osteosarcoma progression and improve patient survival.

Wang et al. (2016) reported that DATS induce apoptosis in osteosarcoma cells through downregulation of the PI3K/Akt pathway mediated by reactive oxygen species. The data of He et al. (2020) demonstrated that DATS exerts the anti-cancer effects by inhibiting cell invasion, migration and proliferation *in vitro* and *in vivo*. The study of Liu et al. (2024) also demonstrated that DATS inhibited the cell growth, migration, EMT and induced apoptosis, autophagy in osteosarcoma cells. Upregulation of CRT expression by DATS may be one of the mechanisms by which DATS inhibits the growth ability of human osteosarcoma Saos-2 cells (Xie et al., 2018). Zhang’s results indicated that DATS inhibits the growth of human osteosarcoma cells by regulating the expression of GRP78 (Zhang et al., 2018b). The relevant data indicated that DATS has significant anti-cancer effects on osteosarcoma cells, and its potential mechanisms include inducing cell apoptosis and reducing multidrug resistance (Wang et al., 2014).

These results provide evidence and strategies for using DATS alone or in combination with standard prevention and treatment of osteosarcoma.

### 2.7 The anti-cancer effect of DATS in liver cancer

Liver cancer is a common malignant tumor in the world, with high incidence rate and mortality. There is no ideal treatment strategy at present. Many researchers have attempted to explore the anticancer effects of natural plant compounds such as DATS in liver cancer (Table 5).

**TABLE 5:** Overview of the effect of DATS on liver cancer and thyroid cancer.

Effects	Target	Subjects	References
Induced pre-apoptotic autophagy	AMPK/SIRT1 pathway	Liver cancer cells	Sun et al. (2022)
Inhibited cell growth	STAT3, Akt, and Erk1/2 pathway	Liver cancer cells	Guan et al. (2022)
Induced cell apoptosis	Several key proteins	Rat hepatic stellate cells	Zhang et al. (2017)
Induces mitochondrial apoptosis and cell cycle arrest	—	Thyroid cancer cells	Zheng et al. (2019)
Inhibited cell growth	NF-κΒ/p65 pathway	Thyroid cancer cells	Xu et al. (2020)
Compromised the stem cell phenotype	AKT/SOX2 axis	Thyroid cancer cells	Zhang et al. (2021)
Induced cell apoptosis	MAPK pathway	Thyroid cancer cells	Pan et al. (2018)

DATS induces pre-apoptotic autophagy in human liver cancer HepG2 cells through the AMPK/SIRT1 signaling pathway (Sun et al., 2022). The results of Guan et al. (2022) indicated that APPL1 polyubiquitination may mediate the inhibitory effect of DAT on HepG2 cell survival by regulating the STAT3, Akt, and Erk1/2 pathways. It is reported that DATS is an inducer of hepatic stellate cells apoptosis, and multiple proteins may be involved in the molecular mechanism of DATS induced cell apoptosis (Zhang et al., 2017).

### 2.8 Thyroid cancer

Thyroid cancer is a common endocrine malignant tumor. The incidence rate of thyroid cancer has been rising. The existing treatment methods are not ideal. There is an urgent need to develop more promising new drugs or strategies to improve treatment efficiency. DATS, as one of the main active ingredients in garlic, is expected to become a new therapeutic strategy for thyroid cancer (Table 5).

The research results of Zheng et al. (2019) revealed that DATS induces mitochondrial apoptosis and cell cycle arrest by triggering DNA damage in anaplastic thyroid carcinoma cells, which may provide a new therapeutic approach for thyroid carcinoma treatment. As a H2 S donor, DATS suppressed the growth of human papillary thyroid cancer cells, inhibited cancer stem cell phenotype and restored thyroid-specific gene expression (Xu et al., 2020; Zhang et al., 2021). DATS induced apoptosis in human papillary thyroid cancer BCPAP cells by activating the MAPK pathway (Pan et al., 2018). The above study elucidates prospective therapeutic targets for thyroid cancer treatment.

### 2.9 Other cancer

In addition to the cancers that have been extensively studied, DATS also has intervention and therapeutic effects in many other cancers.

DATS could affect the apoptosis and immune status of leukemia cells, thereby exerting intervention effects (Suda et al., 2014; Hung et al., 2015). It was reported that DATS can interfere with pancreatic cancer by regulating cell cycle and apoptosis (Ma et al., 2014). Mitochondrial Ca^2+^ overload through voltage gated Ca^2+^ entry contributes to the anti-melanoma effect of DATS (Nakagawa et al., 2020). DATS inhibited the migration, invasion of melanoma cells by inhibiting the integrin/facial adhesion kinase pathway (Wang et al., 2017). DATS mitigated chemotherapy sensitivity of ovarian cancer by regulating the AMPK/SIRT1 pathway (Wang et al., 2023). DATS induced ROS mediated mitotic arrest and cell apoptosis, thereby inhibiting head and neck squamous cell carcinoma tumor growth and tumor stem cell characteristics (Mathan et al., 2024). DATS has inhibitory effects on the tumor biology of synovial sarcoma cells, including triggering cell apoptosis, inducing G2/M cell cycle arrest, increasing intracellular ROS, and damaging mitochondria (Xia et al., 2022). DATS promoted miR-127-3p and inactivates the PI3K/AKT signaling pathway, enhancing the anticancer activity of dexamethasone in multiple myeloma subpopulations of cells (He et al., 2021). DATS induced cell apoptosis through destabilization of TRAF6 by suppressing NF-κB signaling in primary effusion lymphoma (Shigemi et al., 2016). The finding of Wang et al. (2015) demonstrated that DATS inhibits the proliferation of ESCC cells by activating ERK1/2 *in vitro* and *in vivo*. Diallyl trisulfide, resveratrol, and epigallocatechin-3-gallate exerted synergistic inhibitory effects on skin cancer cell lines through the mitochondrial caspase dependent pathway (Arumugam and Alagar Yadav, 2024).

## 3 Discussion, challenges, and future research directions

The prevention and treatment of tumors, especially early prevention and treatment, is a global challenge. Currently commonly used tumor treatment drugs have strong toxicity and adverse reactions to normal cells, which can easily lead to drug resistance. Researchers need to develop and search for drugs with fewer side effects and less susceptibility to drug resistance. Natural plant chemicals are becoming alternative resources for combating cancer due to their unique advantages. Compared with traditional drugs, phytochemicals such as DATS have various advantages, including wide source, easy application, high security and excellent anti-cancer effect. DATS is a natural organosulfur compound derived from garlic that has been investigated as a very promising anticancer drug. DATS has multiple anti-cancer mechanisms, such as regulating immunity, inhibiting proliferation, suppressing migration, triggering apoptosis, and so on. DATS also enhanced the efficacy of traditional anti-cancer drugs, reduced the side effects of anti-cancer drugs and reduced incidence of drug resistance.

The application of DATS in the clinical treatment and prevention of cancers is still greatly limited. There are still many challenges that need to be addressed in order to better apply DATS to early clinical intervention and treatment of cancer. The clear role and precise molecular mechanism of DATS in early intervention and treatment of cancer need to be further explored and elucidated. There is an urgent need to establish methods that can accurately measure the concentration of DATS in blood or target tissues. More clinical and preclinical trials are needed to verify the anti-tumor effect of DATS, or its combined effect with commonly used anticancer drugs. We still need to find better forms of DATS delivery to improve the bioavailability of DATS. Attempts can be made to deliver DATS through exocrine pathways to enhance the bioavailability and targeting of DATS in cancer intervention and treatment. In addition, exosomes derived from plant chemistry from DATS can also be directly extracted, acting on malignant transformed cells, cancer stem cells and cancer cells to observe whether it can enhance the anti-cancer effect and bioavailability of DATS.
